# Societal and individual drivers of fertility desires and intentions among people living with HIV: a cross sectional study of HIV clinic attendees in Soweto, South Africa

**DOI:** 10.3934/publichealth.2022013

**Published:** 2021-12-20

**Authors:** Penelope Chirambira, Sphiwe Madiba, Busisiwe Ntuli

**Affiliations:** Department of Public Health, School of Health Care Sciences, Sefako Makgatho Health Sciences University, Pretoria 0001, South Africa

**Keywords:** South Africa, people living with HIV, life-long ART, fertility desire, intentions, child

## Abstract

**Background:**

High proportion of people living with HIV (PLHIV) who are in the prime of their reproductive years desire to have children. There are limited studies that explore the range of fertility intentions for PLHIV. This study investigated the fertility desires and intentions of PLHIV and the associated factors.

**Methods:**

This was a cross-sectional study of 442 PLHIV receiving antiretroviral treatment (ART) in health facilities in Soweto, an urban township that is situated in the City of Johannesburg in South Africa. STATA version 13 was used to analyze the data.

**Results:**

The participants' mean age was 36.3 years, 70% were females, 79.6% had at least one biological child, and 36% had 3+ children. Almost half (47%) expressed the desire for children, saying that this was because they had no biological children, or their partners wanted children, or they wanted children of a particular sex, or were feeling healthy after taking ART. An increased fertility desire was associated with absence of biological children (AOR = 5.06, 95% CI: 2.11–12.1) and with being married (AOR = 2.63, 95% CI: 1.31–5.27). A decreased fertility desire was associated with being aged 36+ (AOR = 2.63, 95% CI: 1.31–5.27), having primary education (AOR = 0.11, 95% CI: 0.01–1.30) and having ≥4 years of ART duration (AOR = 0.45, 95% CI: 0.24–0.81).

**Conclusion:**

Individual factors played a significant role in shaping the fertility desires of PLHIV in this setting. The high desire for children underscore the need to integrate reproductive health services in HIV and AIDS care and treatment services and develop safer conception programmes to help PLHIV to conceive and have children safely.

## 1. Introduction

Across Southern and sub-Saharan Africa (SSA), the vast majority of people living with HIV (PLHIV) are adults of reproductive age [1]–[3]. Just like the general population, PLHIV desire to have children. After learning of their HIV-status, they desire to start families or to have more children to achieve their desired family size [4]–[8]. In the past, policies in many countries discouraged PLHIV from having children in order to reduce vertical transmission. Consequently, being infected with HIV posed a huge threat to the expression of reproductive desires because of the potential risks of reinfections and the orphaning of existing and future children [8],[9].

Medical achievements in the treatment and management of HIV/AIDS have seen a profound reduction in the level of risk posed for PLHIV by having children. The increased access to antiretroviral therapy (ART) and the availability of prevention of mother-to-child transmission (PMTCT) interventions have changed the patterns of morbidity and mortality globally. With HIV being viewed as a manageable chronic illness, fertility issues in PLHIV have altered in favor of childbearing through PMTCT and safe conception interventions [8]. ART has transformed the general desire to have children into an actual intention [2],[10].

While a strong link has been established between improved health status and reproductive desire among PLHIV, fertility desire can be influenced by a number of factors that operate at the societal and individual levels [8],[11]. At a societal level, socio-cultural expectations play a significant role in shaping the reproductive desires of PLHIV [6],[11]–[15]. For PLHIV, meeting family and societal obligations concerning reproduction may be more important than any risk of HIV transmission that may exist [16]. In SSA, research has documented the enormous pressure on women to have children, regardless of their HIV status [4],[6],[7],[17],[18]. Motherhood is a key component of a woman's identity and is important to her social status [14],[16],[19].

Equally, for men, not having children often results in stigmatization and loss of social status among their peers [20]. Fatherhood is critical, as their perceived masculinity and status in the community depends on their ability to have children to ensure the continuation of the family name and lineage [3],[18]. In many societies in SSA the husband makes decisions regarding the couple's sexual activities, fertility, and use of contraceptives [11],[21]. In these patriarchal societies, married women are often pressurized by their husbands to have children [22]. Not having a male child puts a couple under pressure to continue having children until a male heir is produced [23].

In South Africa the fertility desires of PLHIV, in particular of HIV-positive men, have not been sufficiently studied, yet it seems to be generally accepted that male partners have a strong influence on women's fertility-related desires and decisions [1],[20],[24]. The lack of data might be because of the healthcare seeking behavior of men. Data in South Africa show that health-seeking behavior of men with regards to HIV services is biased towards women, thus more women visit HIV clinics for ART [25]. The purpose of the study was to investigate the fertility desires and intentions of adult PLHIV and determined the associated factors. By including both males and females, the study will bridge the gap on the lack of data on male partners, albeit the number of males who access ART services is small. HIV and reproductive health programs should acknowledge the needs of PLHIV to have children and provide the necessary counseling and other services to support clients to conceive and have children safely [26],[27].

## 2. Materials and method

### 2.1. Study design

A descriptive cross-sectional survey was conducted over a period of four months, from November 2017 to February 2018. The setting for the study was primary health facilities (PHC) in a health sub-district in Soweto, an urban township that is situated in the City of Johannesburg, in South Africa. Soweto is the biggest township in the country with a population of 1,695,047. The population is predominantly black (98.5%), males make up 49.6% of the population while females make up 50.4%. The sub-district where the study was conducted has 23 primary health facilities from which five were selected randomly for the study. All the facilities offer comprehensive primary health services including HIV testing and counseling, and the initiation and continuation of ART treatment for adults and children.

### 2.2. Study population

The population of the study consisted of PLHIV who were receiving ART at facilities in the health district. The participants were selected if they were between the ages of 21 and 49 years for women and 21 and 59 years for men, who visited the selected health facilities for their ART treatment during the period of the data collection. Those who were too ill to be interviewed were excluded from participating in the study. The sample size was determined using the Rao soft sample calculator [28], with a population size of 1500 at a confidence interval of 95% and a margin of error of 5% with a 50% response distribution rate. The sample size required was 369, and with a 20% adjustment to cater for potential refusals, 442 was the total sample size.

### 2.3. Data collection

A structured, piloted, interviewer-administered questionnaire was used to collect data. The questionnaire was developed in English and was translated into two local languages, namely IsiZulu and Sesotho, to cater for the diverse population of the township. The questionnaire was informed by extensive literature review and the objectives of the study [4],[5],[12]. It included demographic information, the period since the HIV diagnosis, the duration on ART, the disclosure of the HIV status to the sex partner, the perceived health status, the reproductive history, and the desire and intention to have children since the subject found out about his/her HIV status. The primary issue to be studied was fertility desire, which was defined as the desire to have children in future.

The participants were recruited in the mornings in the clinics as they came for their scheduled appointments for medication refill or check-ups, and interviews were conducted only after they had done with their business at the clinics. The investigators worked closely with the facility managers, who introduced them to the nurses responsible for HIV treatment and care. The nurses were introduced to the study by being given study information sheets and informed about the inclusion criteria. The facility staff particularly nurses identified patients who met the inclusion criteria and referred them to the researchers. Due to the sensitivity of the study, this recruitment method was appropriate to prevent accidental disclosure of the HIV status of the patients. Patients who met the inclusion criteria were subsequently referred to a room, which had been reserved for completion of the questionnaire in private. The purpose of the study was thoroughly explained to the potential participants, who were informed about voluntary participation, the confidentiality of their responses, and withdrawal from the study at any point without any penalties. Informed consent was then obtained from those who gave their consent to participate before the questionnaire was administered the lead author. There was general willingness to participate in the survey and none of the patients referred to the researcher refused to participate in the study. Although an about 70–100 people visit the different clinics HIV treatment daily, an average of 15–20 questionnaires could be completed per day.

### 2.4. Data analysis

Data were captured into Microsoft Excel spreadsheet before being exported to STATA version 13 for analysis. Descriptive statistics were computed to generate frequencies for all categorical variables, such as gender and marital status. Continuous variables such as age were summarised using means and standard deviation using a t-test. Bivariate analysis was performed between the dependent variable (the desire to have children) and each of the independent variables explored in the study, using Pearson's chi-square test of association. All variables identified to be significant at P < 0.05 were included in the multivariate model to determine the factors associated with fertility desire, using a backward stepwise logistic regression method. Odds ratios (OR) at 95% confidence intervals were obtained, as were p-values.

### 2.5. Ethics approval and consent to participate

The study was approved by Sefako Makgatho University Research Ethics Committee (protocol number: SMUREC/H/58/2017). Permission was granted by the Gauteng Department of Health—Johannesburg Health District (reference number GP_201733_699). All participants gave written informed consent before participation. To ensure anonymity, no personal identifiers were collected, from the participants.

## 3. Results

### 3.1. Description of the study participants

The description of the study participants are presented in Table 1. Of the 442 PLHIV who participated, 309 (70%) were female, their ages ranged from 22 to 57 years with a mean age of 36.3 years (SD ± 7.03 years). With regard to marital status, 20.8% were married, 29.4% were cohabiting with partners in a committed relationship, and 36.7% were single but having a steady sexual relationship. The majority (79.6%) had biological children, 9.7% were raising a child who was perinatally infected with HIV, and 6.6% had lost a child in infancy due to AIDS related illness. The majority (63.1%) had completed high school, 63.8% were employed.

A high proportion (43.4%) had known about their HIV status for four years or more, and 41% had received ART for four years or more. The level of disclosure was high, 86.2% had disclosed to at least one significant person, 69.3% had knowledge of their partners' HIV status. The majority (94.6) perceived their health status to be good.

Concerning fertility intentions, almost have (49%) desired to have a child, 52.2% intended to have one child, 46.4% were already trying for a baby, and over a third (36.8%) intended to have a baby in within the next three years.

**Table 1.** Socio-demographic, clinical, and reproductive characteristics of study participants.

Variables	Frequency	Percent
Gender		
Female	309	69.9
Male	133	30.09
Age Categories		
22–25 years	25	5.7
26–35 years	183	41.4
36–45 years	180	40.7
46 years and above	54	12.2
Level of education		
No formal school	4	0.9
Primary school	81	18.3
High school	279	63.1
Tertiary	78	17.7
Employment status		
Employed	282	63.8
Unemployed	160	36.2
Marital status		
Married	92	20.8
Living together	130	29.4
Single	162	36.7
Divorced	32	7.2
Widowed	26	5.9
Period since HIV diagnosis		
Less than a year	26	5.9
One year	48	10.9
Two years	88	20.0
Three years	88	20.0
Four years and above	192	43.4
Period on ART treatment		
Less than a year ago	43	7.7
One year	57	13.0
Two years	95	21.5
Three years	77	17.4
Four years and above	179	41.0
Disclosed HIV status		
Yes	381	86.2
No	61	13.8
Know partner's status		
Yes	269	69.3
No	119	30.7
Self-reported health status rating		
Good	418	94.6
Poor	24	5.4
Biological children		
Yes	352	79.6
No	90	20.4
Number of biological children		
1	102	29.0
2	123	34.9
3 or more	127	36.1
Has perinatally infected child		
Yes	34	9.7
No	318	90.3
Lost a child to HIV		
Yes	29	6.6
No	413	93.4
Desire to have children		
Yes	209	47.3
No	212	48.0
Not sure	21	4.7
Number of children desired		
1	109	52.2
2	72	4.5
3 and above	28	13.4
When do you desire to have a child?		
Already trying/pregnant	97	46.4
Not yet sure	35	16.8
Within 1-3 years	77	36.8

### 3.2. Factors associated with the desire to have children

In the multiple logistic analysis (Table 2), age, education, and duration on ART were associated with fertility desire. PLHIV older than 36 years (AOR = 0.23, 95% CI: 0.05–0.95; p = 0.043), being older than 46 years (AOR = 0.12, 95% CI: 0.02–0.67, p = 0.016), having primary education (AOR = 0.11, 95% CI: 0.01–1.30, p = 0.026) and being on ART for 4 years and above (AOR = 0.45, 95% CI: 0.24–0.81, p = 0.009) had decreased odds of fertility desire. PLHIV who did not have biological children and those who were married had had increased odd of desiring to have children. Those who had no biological children were 5 times more likely to have fertility desire (AOR = 5.06, 95% CI: 2.11–12.1, p < 0.001) compared to those who had biological children. Married participants were 3 times more likely to have fertility desire than those who were not married (AOR = 2.63, 95% CI: 1.31–5.27, p = 0.006).

**Table 2.** Multivariate logistic regression of factors predicting reproductive desire.

	Total%	Crude ORs	95% CIs	Adjusted ORs	95% CIs
Gender					
Female	69.9	Ref		Ref	
Male	30.09	1.39	0.92–2.11	1.19	0.67–2.10
Age category
22–25 years	5.7	Ref		Ref	
26–35 years	41.4	0.47	0.13–1.67	1.02	0.25–4.17
36–45 years	40.7	0.06	0.01–0.22	0.23	0.05–0.95
46–49 years	12.2	0.01	0.00–0.07	0.12	0.02–0.67
Level of education
No formal education	0.9	Ref		Ref	
Primary	18.3	0.07	0.00–0.77	0.05	0.00–0.71
Secondary	63.1	0.42	0.04–4.14	0.11	0.01–1.30
Tertiary	17.7	0.49	0.04–4.98	0.10	0.00–1.24
Employment status
Not employed	63.8	Ref		Ref	
Employed	36.2	0.92	0.60–1.34	1.41	0.79–2.49
Have biological child
Yes	79.6	Ref		Ref	
No	20.4	8.21	4.28–15.7	5.06	2.11–12.1
Perceived health status
Poor	5.4	Ref		Ref	
Good	94.6	0.52	0.50–2.82	1.86	0.51–6.70
Know partner status
No	30.7	Ref		Ref	
Yes	69.3	3.68	2.26–5.97	1.50	0.80–2.83
Duration on ART
<4 years	59.5	Ref		Ref	
≥4 years	48.5	0.15	0.09–0.23	0.45	0.24–0.81
Marital status
Not married	79.2	Ref		Ref	
Married	20.8	1.77	1.09–2.87	2.63	1.31–5.27

### 3.3. Reasons for the fertility desire of the participants

Almost half (47%) of the participants desired to have children, a high proportion (39%) wanted to try for a child of a particular sex, 23% wanted to try for a boy child, and 11% wanted to try for a girl child, 29.7%) did not have children of their own, and 20% reported that their partners want more children. More women (n = 32) than men (n = 10) would consider child bearing because their partner want more children.

Of the 51% of participants who did not want children in future, the most commonly cited reason being having the desired number of children (42.5%). In addition, 21% indicated that they did not have a stable partner, whereas, 14% felt that they were too old to bear children.

**Table 3.** Reasons for the fertility desire of the PLHIV by gender.

Reason for desiring children	Total	Male n (%)	Female n (%)
Desire to have children	209 (47.3)	72 (54.1)	137 (44.3)
I do not have children of my own	62 (29.7)	24 (33.3)	38 (27.7)
My partner wants more children	42 (20.1)	10 (13.9)	32 (23.4)
I feel healthy due to the ART treatment	41 (19.6)	12 (16.7)	29 (21.2)
I want to try for a boy/girl child	39 (18.6)	15 (20.8)	24 (17.5)
Personal desire to have more children	19 (9.1)	8 (11.1)	11 (8.0)
My child should have siblings	6 (2.9)	3 (4.2)	3 (2.2)
Reasons for not wanting children
Do not desire to have children	212 (48)	58 (43.6)	154 (49.8)
I have enough children	99 (42.5)	32 (52.5)	67 (39)
I am too old	33 (14.2)	7 (11.5)	26 (15.1)
I do not have a stable partner	27 (21.0)	6 (9.8)	21 (12.2)
My partner does not want more children	22 (9.4)	9 (14.8)	13 (7.6)
Fear of compromising my health status	17 (7.3)	0 (0.0)	17 (9.9)
I do not have a stable income	14 (6.0)	4 (6.6)	10 (5.8)
I am too sick to have a child	12 (5.2)	1 (1.6)	11 (6.4)
Fear of infecting my unborn child	6 (2.6)	1 (1.6)	5 (2.9)

## 4. Discussion

The study investigated the fertility desires and intentions of PLHIV on long-term ART in health facilities in Soweto, South Africa. The combined fertility desire was significantly high. We found that almost half (47%) of both the male and the female participants desired to have a child in the future. The combined rate of fertility desire of 47% was higher than those found in Uganda (31%), Tanzania (37%), Kenya (34%), and Malawi (33%) [6],[8],[9],[26]. In contrast, the fertility rate in the current study was lower than those from Uganda (63.1%) and Nigeria (73.2%) [11]. The rate of desired fertility is generally high in SSA because of the context in which PLHIV live and the great value placed on parenthood [8],[12],[21]. The strong fertility desire observed in the current study has public health implications for HIV prevention. Mmbaga et al. [6] argue that strong fertility desire justifies the integration of HIV care and reproductive health so that appropriate services may be offered to PLHIV.

In the current study, the most common cited reasons for wanting children were the lack of existing biological children and a preference for a child of a particular sex. These findings are consistent with those of other studies [6],[18],[29],[30]. Those who did not have children of their own were five times more likely to desire children than those who already had children. Other studies have made similar findings [6],[18],[30]–[32]. The ideal number of children that individuals would like to have is a predictor of fertility desire.

Consistent with past studies in SSA [6],[8],[11],[29], age was a predictor of fertility desire. We found that those older than 35 years had less desire for children than those below 36 years. Decreasing desire for children among older people was reported in several studies [6],[9],[22],[33]. The majority of PLHIV in SSA are in the prime of their reproductive years, therefore, it is natural and to be expected that younger, unmarried PLHIV, would be eager to start families [6],[11]. In addition, those who were married were 3 times more likely to have fertility desire than those that were not married. This is consistent with other studies where those in long term stable relationships considered child bearing [6],[10],[11],[27],[30]. Being married not only creates a sense of security but it provides a reliable support for the raising of children [27]. The PLHIV's desire for children underscores the need for counseling and reproductive services to help PLHIV to conceive and have children safely in the future [30].

Prior studies have found that PLHIV who have attained qualifications were more likely to have fertility desires [32],[33] probably because they might be well-informed about the effects of ART on their health and are also more empowered to make informed decisions about their reproductive choices [32]. We did not find an association with higher qualifications, but we found that those who had primary level education were less likely to express fertility desires. Although the availability of ART has yielded good health outcomes for PLHIV and restored their reproductive desires [8],[10],[34], in this study, those who have been on ART for four years or longer were less likely to express fertility desire. The data showed that most (82%) of them were older than 36 years, an age group that did not desire children.

The results of this survey should be interpreted in the context of the following limitations. We did not collect clinical data such as viral load because of poor record keeping in most facilities, as a result, we could not assess the impact of viral load and CD4 count levels on the desire and intentions to have children, and instead we relied on the participants self-reported health perceptions. Although we wanted to assess the fertility desires of both men and women, the proportion of men compared to women in the facilities were we conducted the survey were small. Literature in South Africa shows that health-seeking behavior of men with regards to HIV services is biased towards women, thus more women visit HIV clinics for ART [25]. Lastly, our use of a convenience sampling technique to select the study participants affected the ability to generalise the findings beyond settings similar to that of the current study. Furthermore, we do not have any data on participants who might have refused participation because we relied on the nurse referring the potential participants to the researchers after consultation. Therefore, those who might have decline participation could have informed the nurse and were subsequently not referred to the researcher for completion of the questionnaire.

## 5. Conclusions

A desire to have children was expressed by almost half of our sample, supporting the view held that an HIV diagnosis does not eliminate a desire for fertility. The findings have implications for the development of guidelines and the design of programs that include discussions about future fertility in HIV counselling.

The study found that a fertility desire was driven mostly by individual factors. Increased fertility desire was associated with the lack of biological children and being married. Older age, low levels of education, longer duration on ART were associated with decreased fertility desire. Although not statistically significant, for female participants, the partner's fertility desire influenced their intentions to have children.

There is a need to integrate reproductive health services in HIV and AIDS care and treatment services and develop programmes to provide safer conception to help PLHIV to conceive and have children safely. While being male was not a predictor of a desire for children, men should be especially targeted by the interventions for safe conception. This underscores the need to design programs that are male-friendly to engage men in safer conception interventions.

## References

[1] Bekker L, Black V, Myer L (2011). Guideline on safer conception in fertile HIV-infected individuals and couples: guideline. S Afr J HIV Med.

[2] Gutin SA, Namusoke F, Shade SB (2014). Fertility desires and intentions among HIV-positive women during the post-natal period in Uganda. Afr J Reprod Health.

[3] Van Zyl C, Visser MJ (2015). Reproductive desires of men and women living with HIV: implications for family planning counselling. Reprod Biomed Online.

[4] Beyeza-Kashesya J, Ekstrom AM, Kaharuza F (2010). My partner wants a child: a cross-sectional study of the determinants of the desire for children among mutually disclosed sero-discordant couples receiving care in Uganda. BMC Public Health.

[5] Cooper D, Moodley J, Zweigenthal V (2009). Fertility intentions and reproductive health care needs of people living with HIV in Cape Town, South Africa: implications for integrating reproductive health and HIV care services. AIDS Behav.

[6] Mmbaga EJ, Leyna GH, Ezekiel MJ (2013). Fertility desire and intention of people living with HIV/AIDS in Tanzania: a call for restructuring care and treatment services. BMC Public Health.

[7] Rispel LC, Metcalf CA, Moody K (2011). Sexual relations and childbearing decisions of HIV-discordant couples: an exploratory study in South Africa and Tanzania. Reprod Health Matter.

[8] Wekesa E, Coast E (2014). Fertility desires among men and women living with HIV/AIDS in Nairobi slums: a mixed methods study. PLoS One.

[9] Krashin JW, Haddad LB, Tweya H (2018). Factors associated with desired fertility among HIV-positive women and men attending two urban clinics in Lilongwe, Malawi. PloS One.

[10] Hussen Mekonnen FE (2017). Effect of antiretroviral therapy on changes in the fertility intentions of human immunodeficiency virus-positive women in Addis Ababa, Ethiopia: a prospective follow-up study. Epidemiol Health.

[11] Matovu JK, Makumbi F, Wanyenze RK (2017). Determinants of fertility desire among married or cohabiting individuals in Rakai, Uganda: a cross-sectional study. Reprod Health.

[12] Adilo TM, Wordofa HM (2017). Prevalence of fertility desire and its associated factors among 15-to 49-year-old people living with HIV/AIDS in Addis Ababa, Ethiopia: a cross-sectional study design. Hiv/aids (Auckland, NZ).

[13] Bakeera-Kitaka S, Nabukeera-Barungi N, Nöstlinger C (2008). Sexual risk reduction needs of adolescents living with HIV in a clinical care setting. AIDS Care.

[14] Mokgatle M, Molapisi E, Madiba S (2017). The childbearing desires of perinatally infected female adolescents enrolled in an HIV clinic in Tshwane District, Gauteng Province, South Africa. Curr Pediatr Res.

[15] Ngure K, Baeten JM, Mugo N (2014). My intention was a child but I was very afraid: fertility intentions and HIV risk perceptions among HIV-serodiscordant couples experiencing pregnancy in Kenya. AIDS Care.

[16] Cooper D, Harries J, Myer L (2007). “Life is still going on”: reproductive intentions among HIV-positive women and men in South Africa. Soc Sci Med.

[17] Ayieko J, Ti A, Hagey J (2017). HIV status and treatment influence on fertility desires among women newly becoming eligible for antiretroviral therapy in western Kenya: insights from a qualitative study. Reprod Health.

[18] Demissie DB, Tebeje B, Tesfaye T (2014). Fertility desire and associated factors among people living with HIV attending antiretroviral therapy clinic in Ethiopia. BMC Pregnancy Childb.

[19] Kaoje A, Ibrahim M, Njoku C (2015). Predictors of fertility desire among people living with HIV attending anti-retroviral clinic in a tertiary health facility in Sokoto, Northern Nigeria. Sahel Med J.

[20] Mantell JE, Smit JA, Stein ZA (2009). The right to choose parenthood among HIV-infected women and men. J Public Health Pol.

[21] Banjo OO, Akinyemi JO (2018). Spousal and household characteristics associated with Women's fertility in sub-Saharan Africa. JPSS.

[22] Haile F, Isahak N, Dessie A (2014). Fertility desire and associated factors among people living with HIV on ART. Harari Regional State Eastern Ethiopia J Trop Dis.

[23] Sofolahan YA, Airhihenbuwa CO (2013). Cultural expectations and reproductive desires: experiences of South African women living with HIV/AIDS (WLHA). Health Care Women Int.

[24] Kawale P, Mindry D, Stramotas S (2014). Factors associated with desire for children among HIV-infected women and men: a quantitative and qualitative analysis from Malawi and implications for the delivery of safer conception counseling. AIDS Care.

[25] Barnabas RV, Szpiro AA, van Rooyen H (2020). Community-based antiretroviral therapy versus standard clinic-based services for HIV in South Africa and Uganda (DO ART): a randomised trial. Lancet Glob Health.

[26] Wagner GJ, Wanyenze R (2013). Fertility desires and intentions and the relationship to consistent condom use and provider communication regarding childbearing among HIV clients in Uganda. Int Scholarl Res Notices.

[27] Ahinkorah BO, Seidu AA, Armah-Ansah EK (2020). Drivers of desire for more children among childbearing women in sub-Saharan Africa: implications for fertility control. BMC Pregnancy Childb.

[28] Raosoft I Sample size calculator, 2004.

[29] Agbo S, Rispel LC (2017). Factors influencing reproductive choices of HIV positive individuals attending primary health care facilities in a South African health district. BMC Public Health.

[30] Shrestha N, Pokharel R, Poudyal A (2020). Fertility Desire and Its Determinants Among People Living with HIV in Antiretroviral Therapy Clinic of Teku Hospital, Nepal. Hiv/aids (Auckland, NZ).

[31] Abebe M, Endazenaw G (2015). Fertility Intention and Family Planning use Among People Living with HIV/AIDS (PLHIV) on Follow up Care Western Shoa Zone, Anti Retroviral Treatment (ART) Unit, Oromia, Ethiopia. AASRJETS.

[32] Tekoh LR, Tesoh AH, Tanjoh NS (2016). Fertility Desire and Reproductive Health Education Needs of Women Living with HIV Receiving Care at Regional Hospital Limbe HIV Treatment Centre. J Adv Med Med Res.

[33] Koyra HC, Biramo YB, Tufa EG (2017). Fertility desire and associated factors among people living with HIV/AIDs at selected health facilities of Wolaita Zone, Southern Ethiopia: cross-sectional study. Am J Public Health Res.

[34] Mujugira A, Heffron R, Celum C (2013). Fertility Intentions and Interest in Early Antiretroviral Therapy among East African HIV-1 Infected Individuals in Serodiscordant Partnerships. J Acq Imm Def Synd.

